# Development of the Teeth

**Published:** 1854-01

**Authors:** 


### ARTICLE XX.

### *Development of the Teeth.*

A very interesting paper on this subject has appeared in the third number of the *Journal of Microscopical Science*, p. 149, from the pen of Mr. T. H. Huxley. Our readers are doubtless aware that the dentine, or proper substance of the tooth, is usually supposed to be formed by ossification of the cells of the pre-existing pulp, and consequently by layers added *within;* while the enamel is considered to be superimposed on it *from without* by a secreting layer, the "enamel organ," folded over the crown of the tooth. This account Mr. Huxley contradicts; the main object of his paper being to prove that the dentine is formed, not by ossification of the pulp, but rather by a process of deposition in, or on its outer layer, and that the supposed "enamel organ" has no function such as that attributed to it, the enamel being separated from it by a membrane (the persistent capsule of Nasmyth) and formed, in some manner not yet explained, on the surface of the crown of the tooth. An analogue of this he finds in the dermic bones of the skate, where there is distinct enamel, and no "enamel organ" whatever; and he brings out the fact by actual observation of the teeth of man.

If a young tooth capsule be opened, it will always be found that a space filled with fluid exists between the inner surface of the capsule and the outer surface of the pulp. The two are perfectly free from all adherence to one another; the only substance between them, besides the fluid, being a more or less abundant whitish matter, which sometimes adheres to the one and sometimes to the other. If the tooth be very young, a structureless membrane may be traced over the whole surface of the pulp; and it is not at all difficult to trace this in perfect continuity on the inner surface of the walls of the capsule.

The whitish substance, just mentioned as lying in the closed sac formed by the membrane so reflected, is delicate, friable, composed of cells, and, in short, is plainly the altered epithelium of the reflected membrane; it is this layer of epithelium which has been dignified with the name of "enamel organ," and invested with the function of secreting or forming the enamel.

Mr. Huxley next proceeds to inquire into the relation of the dental tissues to the tooth-capsule.

If a very young tooth, say from a fœtus of the seventh month, be carefully examined, especially after the addition of acetic acid, under the microscope, the thin cap of tooth-substance may be seen to be everywhere covered by a very delicate membrane, evidently continuous with the reflected capsule described above; and the *enamel fibres* can be distinctly seen *under* this delicate membrane; making it of course obvious, that the so-called enamel organ, being *above* the membrane, can have no such function as that attributed to it. Between the dentine and enamel no trace of membrane can be found.

The next question examined into is that to which we have already alluded—the exact mode of formation of the tooth-substance from the tooth-pulp.

The dental substance, our author holds, is not formed by simple ossification of the cells of the pulp (Nasmyth,) but is deposited within the pulp in definite masses, the gaps between which eventually constitute the dental fibres, *parenchymate materiam suppeditante* (Raschkow.)

When the ossifying boundary of a tooth-pulp is examined, it is seen that where dentification has not begun, the membrane so often mentioned is in immediate contact with the substance of the pulp, which is composed of a homogeneous transparent base, in which closely arranged nuclei are imbedded. Passing towards the ossifying edge, we see in the profile view a clear, more strongly refracting layer, gradually increasing in thickness, which begins to separate the proper substance of the pulp from the investing membrane, and is the young dentine, as transparent as glass, and at first quite structureless in appearance. No trace of "nuclei" can be seen in it; the bodies which have been described as such, being, according to Mr. Huxley, simply lacunæ, and being afterwards found to form the canals of the dentine. He "believes that these facts afford sufficient demonstration that the pulp is *not* converted directly into the dentine; and that the structure of the latter does not depend upon the calcification of pre-existing elements."

In a morphological point of view, the relations of the *cement* show it to be homologous with the enamel. In a very beautiful section of a human tooth from Mr. Busk's cabinet, the upper portion of the cement exhibits in places a very distinct transverse striation, resembling its perfect enamel; and in the tooth of a young calf the transition of the one structure into the other was well shown. The enamel and the cement, therefore, according to Mr. Huxley, are formed on the surface of the dentine, not by the "enamel organ," but in some way which he does not explain. Is it not possible, that not the epithelium, but the very membrane itself, is the agent?

In conclusion: the tooth-pulp being a protrusion of the dermic tissue of the gum, and the capsule an involution of the same, the reflected membrane is the analogue of the basement-membrane of the mucous lining of the mouth, and the "enamel organ" merely its epithelium, inclosed in the sac formed by the involution of the capsule.

The teeth, therefore, are true dermic structures, and are analogous to the hair.—*Amer. Jour. Med. Science.*

